# The effect of combined β-lactoglobulin supplementation and resistance exercise training prior to limb immobilisation on muscle protein synthesis rates in healthy young adults: study protocol for a randomised controlled trial

**DOI:** 10.1186/s13063-023-07329-6

**Published:** 2023-06-13

**Authors:** Alix Hughes, Thomas Francis, Lindsey Marjoram, Jessica H. Rooney, Georgina Ellison-Hughes, Ross Pollock, Michael J. Curtis, Angela Cape, Mads Larsen, Bethan E. Phillips, Philip J. Atherton, Kenneth Smith, Oliver C. Witard

**Affiliations:** 1 Centre for Human and Applied Physiological Sciences, King’s College London, London, UK; 2 School of Cardiovascular Medicine & Sciences, King’s College London, London, UK; 3 Clinical Trials Unit, King’s College London, London, UK; 4 Arla Foods Ingredients, Aarhus, Denmark; 5 School of Medicine, University of Nottingham, Nottingham, UK

**Keywords:** Prehabilitation, Milk protein, Myofibrillar protein synthesis, Muscle mass, Muscle disuse

## Abstract

**Background:**

The decline in skeletal muscle mass experienced following a short-term period (days to weeks) of muscle disuse is mediated by impaired rates of muscle protein synthesis (MPS). Previous RCTs of exercise or nutrition prehabilitation interventions designed to mitigate disuse-induced muscle atrophy have reported limited efficacy. Hence, the aim of this study is to investigate the impact of a *complex* prehabilitation intervention that combines β-lactoglobulin (a novel milk protein with a high leucine content) supplementation with resistance exercise training on disuse-induced changes in free-living integrated rates of MPS in healthy, young adults.

**Methods/design:**

To address this aim, we will recruit 24 healthy young (18–45 years) males and females to conduct a parallel, double-blind, 2-arm, randomised placebo-controlled trial. The intervention group will combine a 7-day structured resistance exercise training programme with thrice daily dietary supplementation with 23 g of β-lactoglobulin. The placebo group will combine the same training programme with an energy-matched carbohydrate (dextrose) control. The study protocol will last 16 days for each participant. Day 1 will be a familiarisation session and days 2–4 will be the baseline period. Days 5–11 represent the ‘prehabilitation period’ whereby participants will combine resistance training with their assigned dietary supplementation regimen. Days 12–16 represent the muscle disuse-induced ‘immobilisation period’ whereby participants will have a single leg immobilised in a brace and continue their assigned dietary supplementation regimen only (i.e. no resistance training). The primary endpoint of this study is the measurement of free-living integrated rates of MPS using deuterium oxide tracer methodology. Measurements of MPS will be calculated at baseline, over the 7-day prehabilitation period and over the 5-day immobilisation period separately. Secondary endpoints include measurements of muscle mass and strength that will be collected on days 4 (baseline), 11 (end of prehabilitation) and 16 (end of immobilisation).

**Discussion:**

This novel study will establish the impact of a bimodal prehabilitation strategy that combines ß-lactoglobulin supplementation and resistance exercise training in modulating MPS following a short-term period of muscle disuse. If successful, this complex intervention may be translated to clinical practice with application to patients scheduled to undergo, for example, hip or knee replacement surgery.

**Trial registration:**

NCT05496452. Registered on August 10, 2022.

Protocol version: 16-12-2022/1

**Supplementary Information:**

The online version contains supplementary material available at 10.1186/s13063-023-07329-6.

## Background

The term *muscle disuse* refers to a scenario in which a muscle group(s) is unloaded over an extended (> 2 days) time period, e.g. limb immobilisation due to injury or following surgery. A period of limb immobilisation-induced muscle disuse is widely established to cause muscle atrophy [8]. In this regard, only 5 days of leg immobilisation was sufficient to elicit a physiologically relevant (3.5%) decline in quadriceps muscle CSA [32]. At the metabolic level, this muscle atrophy is primarily mediated by a decreased response of muscle protein synthesis (MPS) rather than increased stimulation of muscle protein breakdown [24]. Accordingly, a study by the same group demonstrated an ~ 30% decline in response of MPS to protein ingestion after 14 days in a leg brace, indicating that immobilisation led to a state of muscle anabolic resistance [33]. These findings highlight the importance of targeted, non-pharmacological, interventions to attenuate the immobilisation-induced decline in MPS in order to maintain muscle mass during unplanned (i.e. injury) or planned (i.e. surgery) periods of disuse.

Resistance exercise training serves as the most potent non-pharmacological stimulus of MPS [4]. Moreover, the provision of dietary amino acids in combination with resistance exercise exhibits an additive effect, stimulating MPS to a greater degree than exercise alone [5]. Multiple clinical trials have investigated the efficacy of a nutritional intervention to mitigate the suppressed response of MPS during a period of immobilisation [34], However, to our knowledge, no studies to date have investigated the impact of a combined nutritional and exercise prehabilitation programme, implemented prior to a period of immobilisation, on mitigating the disuse-induced decline in MPS and muscle mass. To fill this gap in knowledge, we will conduct a placebo-controlled clinical trial that combines β-lactoglobulin supplementation as a novel milk protein with resistance exercise training and measure integrated free-living rates of MPS before and after 5 days of limb immobilisation. The placebo group will be an energy-matched carbohydrate (dextrose) control.

The biological rationale for administering β-lactoglobulin supplementation in the present study is underpinned by two primary lines of evidence. First, β-lactoglobulin exhibits an exceptionally high leucine content (16%) that exceeds the constituent leucine profile (~ 12%) of whey protein [21]. In addition to providing substrate for the synthesis of new muscle protein, leucine also acts a signalling molecule to trigger MPS via activation of the mechanistic target of rapamycin complex 1 (mTORC1) pathway [1, 2]. Accordingly, several studies have reported a greater acute response of MPS to ingestion of a leucine-rich intact whey protein bolus vs. a dose-matched soy or casein protein bolus that exhibits a lower leucine content [9, 29]. Notwithstanding, it is plausible that a ‘ceiling effect’ may exist regarding leucine availability and the postprandial stimulation of MPS, as evidenced by our previous observation of a plateau in the dose–response of MPS to ingested whey protein in healthy young men [36]. Although a strong positive correlation (*r* = 0.66) between peak plasma leucine concentrations (manipulated by ingestion of intact whey or casein protein) and postprandial rates of MPS rates has been reported in a relatively larger cohort (*n* = 48) of older men [23], this observation has not been reported in healthy young adults and/or in response to a catabolic period of immobilisation.

A secondary rationale for β-lactoglobulin supplementation relates to the marked insulinotropic response and associated direct and/or indirect action of insulin on MPS. In this regard, the serum insulin AUC following β-lactoglobulin ingestion was 62% higher than casein and 30% higher than whey and corresponded with a higher plasma glucose-dependent insulinotropic peptide response [21]. In the presence of exogenous amino acids, insulin has been shown to stimulate MPS via the direct activation of mTORC1 signalling [6, 16, 24]. Insulin also stimulates endothelial-dependent vasodilation and microvascular recruitment [31] via activation of endothelial nitric oxide synthase (eNOS) [28], leading to an increased nutritive (insulin and amino acids) flow to skeletal muscle [15, 30]. Hence, via direct or indirect actions of insulin on MPS, the GIP-dependent insulinotropic properties of β-lactoglobulin serves as an alternative mechanism that may underpin the increased stimulation of MPS during a period of muscle disuse. Therefore, the objectives of this study are to:

1. Establish whether combined β-lactoglobulin supplementation and resistance training for 1 week prior to 5 days of limb immobilisation will attenuate the decrease in integrated free-living rates of MPS during short-term muscle disuse
2. Establish whether combined β-lactoglobulin supplementation and resistance training for 1 week prior to 5 days of limb immobilisation will attenuate the decrease in leg lean mass, muscle fibre cross-sectional area and quadriceps muscle strength during short-term muscle disuse

## Methods/design

### Experimental design

**Fig. 1:**
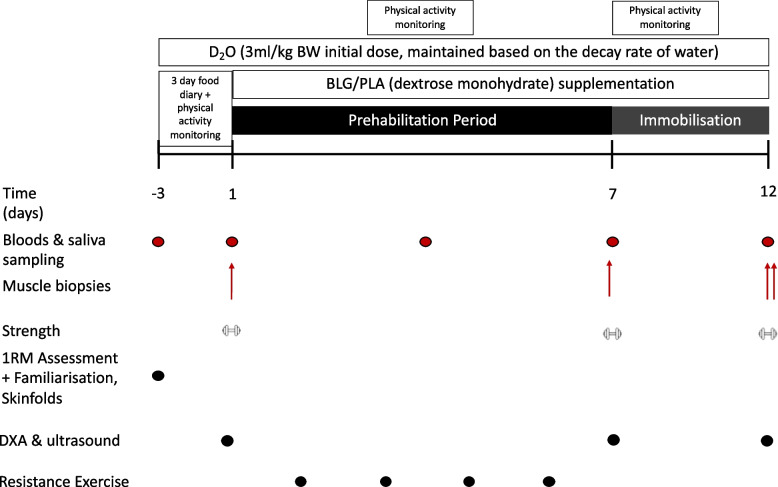
Schematic diagram of experimental protocol. PA, physical activity monitoring via actigraphy. D_2_O, heavy water ingestion period. BLG, β-lactoglobulin supplementation, at 23 g three times per day. PLA, placebo supplementation, energy matched to the β-lactoglobulin supplement. Strength, isometric and isokinetic contractions assessed via isokinetic dynamometry

The aim of this study is to investigate the impact of a *complex* prehabilitation intervention that combines β-lactoglobulin supplementation with resistance exercise training on disuse-induced changes in MPS, leg lean mass, muscle thickness, muscle fibre cross-sectional area and quadriceps muscle strength in healthy, young adults. To address this aim, we will conduct a parallel, double-blind, 2-arm, randomised placebo-controlled trial. An overview of the trial design is displayed in Fig. 1 and detailed in chronological order under the ‘Experimental protocol’ section. The intervention group will combine a structured resistance exercise training programme with thrice daily dietary supplementation with 23 g of β-lactoglobulin. The placebo group will combine the same training programme with an energy-matched carbohydrate (dextrose) control. The study protocol will last 16 days. Day 1 will be a familiarisation session and days 2–4 will represent the ‘baseline’ period. Days 5–11 represent the ‘prehabilitation period’ whereby participants will combine resistance training with their assigned dietary supplementation regimen. Days 12–16 represent the muscle disuse induced ‘immobilisation period’ whereby participants will have their leg immobilised in a brace for 5 days and continue their assigned dietary supplementation regimen only (i.e. no resistance training). The primary endpoint of this study is the measurement of free-living, integrated rates of muscle myofibrillar protein synthesis (myo-MPS) that will be calculated at baseline, over the 7 day prehabilitation period and the 5 day immobilisation period separately. The trial will be parallel in design given that recruited participants must be tracer naïve for the valid determination of MPS rates. Secondary endpoints include measurements of leg lean mass, muscle thickness, muscle fibre cross-sectional area and quadriceps muscle strength that will be collected at baseline (i.e. prior to intervention), after prehabilitation (day 11) and following 5 days of immobilisation (day 16).

### Participants

To address the study objectives, a cohort of 24 healthy young (18–45 years) males and females who satisfy the inclusion and exclusion criteria outlined in Table 1 will be recruited for this trial. Eligibility will be determined using a series of self-report questionnaires and a 3-day weighed food diary to assess baseline dietary protein intake. Volunteers that report a baseline protein intake in excess of 1.2 g·kgBM^−1^·day^−1^ will be deemed ineligible for participation in this study. The protocol for this study has received ethical approval from King’s College London Research Committee (reference: HR/DP-21/22-29290) and will be conducted in accordance with the Human Tissue Act and the Declaration of Helsinki and has been registered at clinicaltrials.gov under reference number NCT05496452 (version: 1, date: 15 December 2022). Participants will be reimbursed £500 for their time invested in the study.

**Table 1:** Inclusion and exclusion criteria

**Inclusion**	**Exclusion**
1. Male and female	1. Dairy allergy or intolerance
2. Aged 18–45 years	2. Lower limb injury or surgery in the last 6 months
3. Healthy	3. Lower limb osteoarthritis or other musculoskeletal disorder
4. Physically active (≥ 150–300 min of moderate-intensity aerobic physical activity per week OR 75–150 min of vigorous-intensity aerobic physical activity per week)	4. A musculoskeletal or blood blotting disorder
5. Eumenorrheic and not taking any hormonal birth control (females)	5. An allergy to local anaesthetic
	6. Currently pregnant
	7. Current use of blood thinning medications
	8. Volunteers that take part in structured resistance exercise training
	9. Taking supplements considered to be anabolic to skeletal muscle (protein supplements, creatine, or omega-3 supplements)
	10. Consuming more than 1.2 g of protein per kilogramme of body mass per day in their habitual diet
	11. Volunteers involved in other studies at the time of enrolment
	12. Volunteers who have taken part in a tracer study in the past 18 months
	13. If without an understanding of verbal or written English
	14. Volunteers with a history of eating disorders

#### Power calculation

The sample size for this study has been estimated using an a priori power analysis of a previously published study by Kilroe et al. [19] that utilised the same unilateral leg immobilisation protocol, primary endpoint measurement (free-living integrated MPS rates assessed by deuterium oxide tracer methodology) and participant characteristics (healthy young adults). We calculated an effect size (d) of 1.6 for the primary endpoint (MPS) using Eq. 1.

1 $$\mathbf d\boldsymbol=\boldsymbol(\mathbf\mu\mathbf1\boldsymbol-\mathbf\mu\mathbf2\boldsymbol)\boldsymbol/\mathbf{SD}$$

where μ1 is the mean value for muscle protein synthesis in group 1, μ2 is the mean value for muscle protein synthesis in group 2 and SD is the pooled standard deviation.

This effect size was entered into G*Power (version 3.1.9.2), with power set to 0.8 and significance level (*α*) set to 0.05. The required sample size was projected to be 12 per group, for a total of 24 participants. Hence, we plan to recruit 24 healthy, young (18–45 years) males and females from the local area. Recruitment will proceed on a rolling basis until the adequate sample size is reached. Emphasis will be placed on considering the demands of the study before enrolment to minimise loss to follow-up.

#### Randomisation process

Eligible participants will be allocated to one of two parallel intervention arms in a 1:1 allocation ratio using a stratified randomisation scheme. A randomisation spreadsheet will be prepared by the named statistician (MC) that enables participants to be stratified by sex as well as treatment. The spreadsheet will contain two randomised sequences, with one for male participants and one for female participants. Since there is only one intervention and its control, meaning there are only two primary groups, we will employ two parallel randomisation sequences, switching between the two based on the sex of each consecutive volunteer. The randomisation is restricted to preclude clustering (a consecutive sequence of the same intervention which, in a two-group study, we refer to as groups ‘A’ and ‘B’). This is important because unrestricted randomisation can generate sequences of consecutive As or Bs which, whilst adhering to a randomisation pattern, would run the risk of introducing confounds. This is because a limited number of participants can be studied at any one time. A sequence of several As and several Bs could result in As or Bs predominately participating in the summer, for example. This is obviated by limiting any consecutive sequence of either intervention to a maximum of two. The randomisation sequence generated for the male spreadsheets is the inversion of that for the female (if the male table sequence starts A, B, B, A, then the female table will start B, A, A, B, and so on).

Unblinded researchers (AC, LM and JR) are responsible for packaging the sachets. LM and JR will provide blinded participant supplies to the study team. The unblinded individuals will not have any involvement in participant management, study related tests, or assessments. Sachets will be packaged into boxes of active or placebo product based on the treatment arm codes provided by the supplier (Arla Food Ingredients Ltd). The unblinded researcher will decide whether ‘A’ or ‘B’ denotes the test drink or placebo and will relabel each box as ‘A’ or ‘B’. When a participant is randomised to the study, the named unblinded researcher will dispense the assigned pack ‘A’ or ‘B’ based on the randomisation list and record the participant ID, participant name and date of supply against the record on the spreadsheet. The ‘A’ or ‘B’ label, as applicable, will be removed from the box by the unblinded researcher and the box code (taken from the randomisation list) and participant ID will be handwritten on the outer box label prior to handing over the supplies to the study team. The A/B codes and randomisation spreadsheet will be stored electronically as a password protected file by the unblinded researcher.

### Experimental protocol

**Fig. 2:**
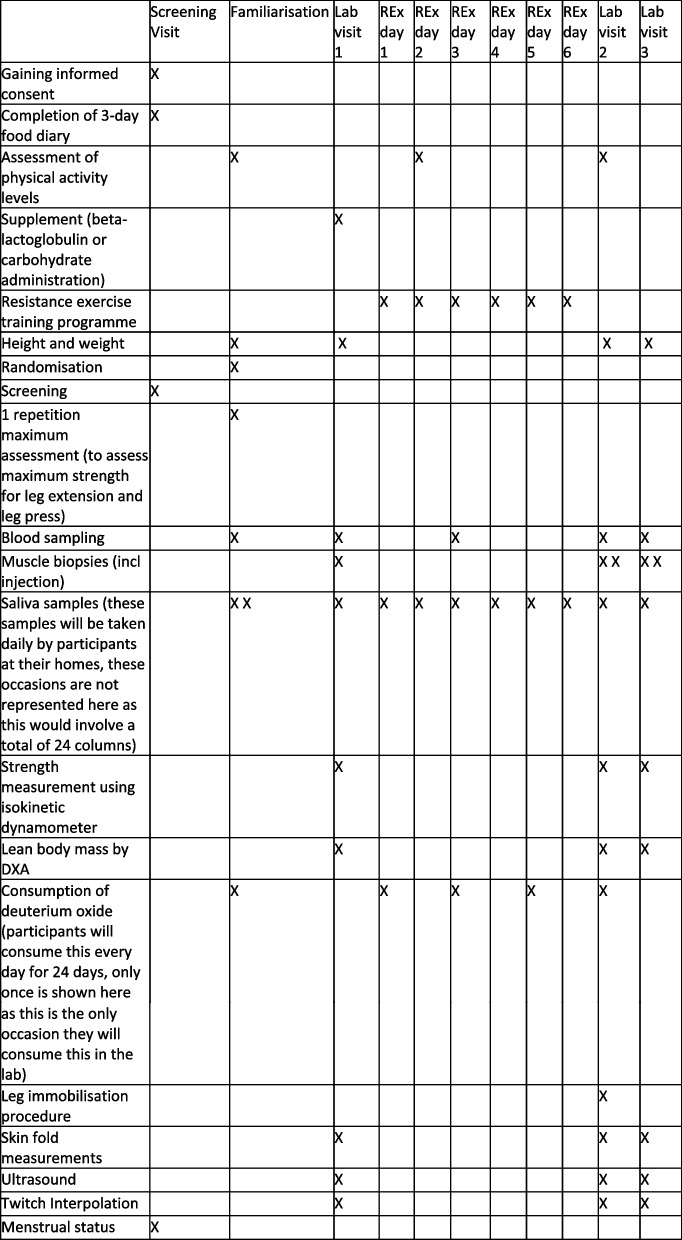
SPIRIT figure showing an overview of the assessment schedule at baseline and follow-up in the study

A SPIRIT figure that details the time points for assessment and intervention is displayed in Fig. 2, and the accompanying SPIRIT checklist is found in Additional file 1.

#### Participant screening

Prospective participants will read the participant information sheet (Additional file 2) and discuss the study with a member of the research team (AH). Next eligibility will be assessed using self-report questionnaires and a 3-day diet diary. If deemed eligible for the study, participants will be guided through the process of written informed consent (Additional file 3). Following consent, participants will be assigned the next Participant ID number and the appropriate treatment arm, by the unblinded study team, in accordance with the randomisation list. Eligible participants will then undergo the trial protocol. All laboratory sessions will be completed at King’s College London (London, UK), beginning with a familiarisation session. Sample analysis will be conducted at the University of Nottingham (Nottingham, UK) and King’s College London (London, UK).

For all female participants, length of the menstrual cycle will be assessed via the calendar method on three separate occasions: one full cycle immediately prior to testing, one full cycle during testing, and one full cycle immediately after testing has been completed. These data will be used to inform the start date of testing for all female participants, as all participants will aim to begin their first laboratory visit at the start of their follicular phase. It also will be used to calculate which phase of their cycle they were in at different points of testing. Menstrual tracking will only take place on one occasion prior to the study starting.

#### Familiarisation

After informed consent has been obtained, participants will visit the Exercise Physiology laboratory at King’s College London 4 days prior to beginning the prehabilitation period to undertake a familiarisation session. After consent has been received, participants will provide a baseline blood and saliva sample. Next, measurements of height and body weight will be obtained, and participants will be provided with an initial D_2_O bolus to consume in the laboratory (see Stable isotopic tracer methodology section for more information). Participants will be familiarised with knee extensor and knee flexor maximal voluntary contractions and the twitch interpolation protocol to assess levels of voluntary activation for the measurement of isometric strength on an isokinetic dynamometer. For this test, with the leg fixed at 90° of flexion, participants will be asked to perform 3 maximal effort quadriceps and hamstring contractions, each held for 3 s, to determine isometric strength. Prior to each quadriceps contraction, the quadriceps muscle will be electrically stimulated to elicit a maximal twitch. The twitch will be delivered during the maximal contractions at all levels of voluntary activation to be assessed via the twitch interpolation technique. Participants also will be familiarised with the isokinetic dynamometry protocol, which involves maximal quadriceps and hamstrings contractions under dynamic conditions. Participants will be asked to contract their muscle to bring their leg from a 90° angle to straight and back again at two different speeds (60°/s and 120°/s).

At this laboratory visit, participants also will complete a one repetition maximum (1RM) assessment for leg press and single leg extension on both legs individually. Once the familiarisation and 1RM assessments have been completed, and 2 h have elapsed, participants will be asked to provide another blood and saliva sample. Finally, participants will be given an activity monitor (Actigraph GT9X) and physical activity questionnaire [7] to take home and wear for 4 days prior to the next laboratory visit (see Diet and physical activity tracking sub-heading). Participants also will be provided daily top-up boluses of D_2_O to consume over the 16-day period and be provided with tubes to obtain daily saliva samples throughout the trial period.

#### Baseline period

On the morning of day 4 (i.e. following 1 day of familiarisation and 3 days of ‘baseline’), participants will undergo an ultrasound of the *rectus femoris* for measurement of muscle thickness and then be assessed for strength using isokinetic dynamometry. Finally, a muscle biopsy will be obtained from the *vastus lateralis* of one leg under local anaesthesia with the participant fasted. This involves a small piece of muscle tissue being extracted (see the ‘Muscle biopsies’ section) and allows for the measurement of integrated MPS over the baseline period.

#### Prehabilitation period

Days 4–11 will represent the ‘prehabilitation period’ and consist of a complex intervention that combines resistance exercise training with nutritional supplementation. The resistance training programme has been modified from the protocol described by Smeuninx et al. [27]. Participants will complete 4 sessions of bilateral resistance exercise training on leg press and leg extension machines during the prehabilitation period. The final bout of resistance exercise will be conducted ≥ 24 h prior to the 5 day immobilisation period. Each session will consist of two warm-up sets at 50% of 1RM, followed by six sets at 75% of their 1RM for both the single leg press and leg extension. Sets will consist of a target 12 repetitions and will be separated by 2 min rest. All training sessions will last ~ 30 min and will be conducted at a time convenient for the participant.

Participants also will receive a milk-based (β-lactoglobulin) or a carbohydrate supplement (dextrose monohydrate) 3 times per day over the prehabilitation and immobilisation periods. In total, the participant will consume 36 drinks over the 12-day period. Lacprodan® BLG-100 Acidic is a pure β-lactoglobulin derived from whey that delivers a unique amino acid profile and 4% more leucine compared to whey protein. The product is manufactured and packaged according to the relevant EU-regulations for food and food ingredients and/or FAO/ WHO Codex Alimentarius to ensure the milk/milk constituents used as raw material originate from healthy cows. The milk used in production is included in monitoring programmes for undesirable substances as required by regulations or HACCP-based risk assessment.

The placebo (dextrose monohydrate) will be modified to match the same flavour, colour and texture of β-lactoglobulin. The dextrose placebo will undergo the same microbiological testing as the β-lactoglobulin to ensure it is safe for consumption. The list of ingredients for dextrose is as follows: dextrose monohydrate, sucralose, citric acid anhydrous, pineapple flavouring (SC894020), lemon flavouring (SBD 213841) and colour (Yellow 410-WS-P). Both products will be stored at room temperature in a cool, dry place. Compliance will be monitored by requesting participants return all empty packaging.

On day 12, participants will undergo the same procedures (ultrasound, isokinetic dynamometry, and muscle biopsy) conducted at ‘Baseline’.

#### Immobilisation period

Days 12–16 represent the ‘immobilisation period’. On day 12, immediately following the muscle biopsy procedure, participants will have one leg immobilised. The chosen leg will be randomly selected such that 50% of participants have their dominant leg immobilised and 50% their non-dominant leg immobilised. Immobilisation will be achieved by fixing a brace around the knee and maintaining 90° of knee flexion for 5 days. A boot will also be placed on the foot to fix the ankle joint at a 90° angle. Participants will be instructed on the use of crutches for ambulation during this period. Anti-tamper tags will be placed on the brace and bootstraps to indicate if the brace/boot has been modified during the immobilisation period. On day 16, the brace will be removed from the leg, a final ultrasound and strength assessment will be conducted, and two more muscle biopsies (one from each leg) will be collected.

### Experimental procedures

#### Stable isotopic tracer methodology

Free-living integrated rates of MPS will be measured using deuterium oxide (D_2_O) tracer methodology [35]. The D_2_O dosing protocol will consist of a loading day and 15 maintenance days. On day 1 of the trial, and having already provided a background saliva sample, participants will consume a loading dose of 70% D_2_O (Sigma-Aldrich, Poole, UK) that is equal to 3 mL/kg body mass with the aim to label the body water pool to ~ 0.3 atom per cent excess (APE). The loading dose will be dispensed into 50 mL doses and consumed at 30 min intervals to reduce risk of nausea and light-headedness associated with disrupted inner ear equilibrium. Body water enrichment will be maintained in a steady state using daily top-ups of D_2_O based on an 8% per day decay rate. Participants will be instructed to provide a daily saliva sample upon waking, followed by consumption of the D_2_O maintenance dose. The study team will filter the heavy water through a 0.22-μm sterile filter before aliquoting the participant specific volumes into sterile bottles.

#### Muscle biopsies

A total of 4 biopsies per participant will be collected from the *vastus lateralis*. Participants will rest in a semi-supine position whilst a 3–5 mm skin incision is made under local anaesthetic (2% lidocaine) using a surgical blade. The percutaneous needle biopsy technique with applied suction [3] will then be used to obtain 300–400 mg of wet muscle tissue from this site with participants in an overnight (> 10 h) fasted state. The skin will be closed with steri-strips and a waterproof dressing applied to keep the site clean. Muscle biopsies will be conducted on 3 laboratory visits. At baseline, and on day 12, a single biopsy will be obtained from the leg randomly assigned for immobilisation in the leg brace and boot. Biopsy 2 will be conducted > 1 cm proximal to biopsy 1. On day 16 (end of immobilisation period), muscle biopsies will be collected from both immobilised and control legs. Tissue will be immediately snap frozen in liquid nitrogen before all tissue will be transferred to the −80 freezer for storage until further analysis of integrated free-living rates of MPS Wilkinson et al. [35] and static markers of muscle protein breakdown [22]. Approximately 50 mg of muscle tissue will be separated to be mounted for histological analysis, which will not be immediately snap frozen. Fibre orientation of this tissue sample will be assessed using a stereomicroscope before mounting on cork with optimal cutting temperature compound with fibres oriented in the transverse plane. These blocks will be immediately frozen in dry ice cooled isopentane and stored at –80 °C until analysis. A cryostat chilled to –20 °C will be used to cut 10 μm serial sections of these histology blocks, which will be used for ATPase and fluorescence immunohistochemistry staining. All muscle, blood and saliva samples will be logged in eLabJournal.

#### Blood and saliva sampling

All blood samples will be obtained from a forearm vein using venepuncture technique with participants in an overnight (> 10 h) fasted state. Samples will be collected into vacuumed EDTA, lithium heparin and serum separator tubes, centrifuged at 3000 × g for 10 min at 4 °C and aliquoted as plasma (EDTA) or serum across five 1.0 mL Cryovial tubes and stored at −80 °C until analysis of deuterium enrichment, amino acid concentration, insulin concentration and urea concentration. Saliva samples will be collected on multiple for the assessment of total body water enrichment. The measurement of total body enrichment is required to determine the deuterium in the body for the calculation of Myo-MPS. Participants will collect a 2-mL saliva sample in a designated vial using the passive drool technique before initial storage in the fridge (at home) and subsequent transfer to −80 °C freezer in the laboratory until subsequent analysis.

#### Body composition

Body mass will be assessed to the nearest 0.1 kg using digital scales (TFW150, Adam Equipment, Milton Keynes, England) with participants wearing identical light clothing at baseline, post prehabilitation and post immobilisation laboratory visits. At baseline, skinfold measurements of body fat percentage will be conducted in duplicate across eight body sites (triceps, biceps, subscapular, iliac crest, supraspinal, abdominal, thigh and calf) by a trained ISAK practitioner using Harpenden callipers (HaB, UK). Body fat will be estimated using the anthropometric equation presented by Eston et al. [13]. Leg lean mass (immobilised and non-immobilised legs) will be measured at baseline, after the 1 week prehabilitation period and after the 5 day prehabilitation period using dual energy x-ray absorptiometry (DXA, Hologic Horizon W scanner). Ultrasound will be used to assess muscle thickness of the *rectus femoris* for both the immobilised and non-immobilised legs at the same time points. For this assessment, leg length from the hip crease to the top of the patella will be measured with the participant seated on a plinth with their legs straight out in front of them. Once this distance has been obtained, 1/3rd of the distance from the patella will be marked on the thigh. A linear ultrasound probe coated in imaging gel will be placed at this position and the *rectus femoris* located. Once located, an image will be taken with the thickest section of this muscle in the centre, from which muscle thickness will be measured.

#### Isokinetic dynamometry

For isokinetic dynamometry, participants will be seated on a Kin-Com (Chattangooga Group) so that their knee is ~ 2 cm away from the edge of the seat and their back is upright and supported by the backrest. Straps will be placed across their chest and waist to isolate movement to the leg only. The lever arm will be positioned to align with the centre of rotation of the knee, and the ankle strapped on to the lever arm ~ 3 cm proximal to the ankle joint. The length from the centre of rotation of the lever arm to the attachment point at the ankle will be noted. Participants will then be asked to perform three single leg maximal contractions at 60°/second, moving their leg from a 90° angle to straight and back again. Participants will be asked to perform three more maximal contractions at 120°/s. Finally, participants will be asked to perform three maximal isometric contractions, with their leg fixed at a 90° angle and the twitch interpolation protocol described above will be applied. Isokinetic dynamometry will be conducted on both the immobilised and non-immobilised legs separately.

#### Diet and physical activity tracking

To assess physical activity patterns, participants will complete a three-day physical activity questionnaire and wear an Actigraph GT9X monitor (ActiGraph, Pensacola, FL, USA) for four consecutive days. These two activity monitoring techniques require participants to include two weekdays and one weekend day. This assessment will be conducted at three different time points throughout the study, once at baseline, once during the resistance exercise training period and once during the immobilisation period, in order to track changes in physical activity at different phases of the study.

The Actigraph will be secured in a pouch and worn on whichever ankle will not be immobilised. Participants will be instructed to remove the monitor during bathing, swimming, and sleeping activities but to wear it at all other times. The monitor records acceleration in three dimensions, and data will be presented as both mean vector magnitude counts per minute (VM) and time spent performing different activity levels. The threshold for these activity levels is based on a validation study for moderate and vigorous activities with this specific monitor [26], in combination with the Actigraph recommended cut point for sedentary activity [14]. Based on these studies, the cut points for sedentary, light, moderate and vigorous activity levels are < 100, 100–10,431, 10,432–19,813, and > 18,914 VM, respectively.

The physical activity questionnaire was selected from Bouchard et al. [7] as a 3-day questionnaire validated in a general population. In brief, the questionnaire requires participants to qualify the type of activity performed during each 15-min period for 72 h on a scale from 1 to 9. The scale will be discussed with participants before they are given the questionnaire. If a participant is uncertain about a particular activity, this will be discussed with a researcher to come to an agreement about the rating. The corresponding energy cost of each category was also taken from Bouchard et al. [7] and will be used to calculate total energy expenditure in METS for the three consecutive days. Dietary energy and macronutrient intake will not be strictly controlled during the trial but will be monitored by the research team to ensure background protein intake does not surpass 1.2 g•kg body mass^−1^•day^−1^.

### Statistical analysis strategy

All statistical analyses will be performed using IBM SPSS Statistics for Windows, v.22.0 (IBM, Armonk, NY, USA). Data will be checked for normal distribution using the Shapiro-Wilk test. The primary endpoint measurement is myo-MPS. Secondary endpoints are leg lean mass (DXA), *rectus femoris* muscle thickness (ultrasound), muscle fibre CSA (immunohistochemistry) and quadriceps muscle strength (isokinetic dynamometry). All measurements will be analysed using a 2-way between and within mixed model ANCOVA with condition (ß-lactoglobulin vs. placebo) as the between-group factor and time period (basal *vs*. prehabilitation *vs.* immobilisation) as within-group factors. Diet and physical activity status will be input as covariates. Where appropriate (i.e. measurements of MPS, leg lean mass, muscle thickness and quadriceps muscle strength), a within-subject control (non-immobilised leg) will be applied to the statistical model when making between group comparisons. The key objective is to compare the effect of the intervention vs. placebo in an unpaired sample. We plan to make three comparisons between the two groups (Δ baseline to immobilisation, Δ prehabilitation to immobilisation, and Δ baseline to prehabilitation for all measurements), disregarding sex as a factor. There is a risk this may introduce type 2 error (rather than type 1 because the randomisation is granular), but we have no reason to anticipate that sex will be a significant confounder. Additional justification for this strategy is because we anticipate that the number of male and female participants will not be equal. The strategy will be to determine from the ANCOVA above whether the intervention is a significant source of variance for each of the primary and secondary endpoint measurements. Bonferroni post hoc testing will be undertaken only if *F* is significant (*P* < 0.05), and there is no variance in homogeneity. The post hoc test will be a *t* test but with an adjustment in the *P* value to accommodate the number of comparisons. The adjustment will be to add a degree of freedom for each of the comparisons. We consider this statistical approach optimal for permitting reliable discovery in a reasonable timeline. Statistical significance will be set an *α*-level of *P* < 0.05.

## Discussion

Previous studies have reported mixed findings regarding the effectiveness of non-pharmacological prehabilitation strategies to mitigate impairments in MPS in response to short-term muscle disuse. For instance, McGlory et al. reported higher integrated rates of myo-MPS following 2 weeks of leg immobilisation when healthy, recreationally active females undertook a 6-week prehabilitation strategy of omega-3 fatty acid supplementation compared to a sunflower oil placebo control [20]. In contrast, thrice daily high dose (15 g/d) leucine supplementation during 7 days of leg immobilisation failed to mitigate the disuse-induced decline in myo-MPS in healthy young males [12]. Moreover, 7 days of resistance exercise prehabilitation failed to attenuate the decline in myo-MPS elicited by 5 days of inpatient bedrest in older adult males [27]. To date, studies of prehabilitation strategies conducted within the context of skeletal muscle disuse have primarily been unimodal in design, focussed on the independent effects of a nutritional intervention [10-12, 17] or exercise-based intervention [18, 27] alone. To our knowledge, the present study is novel in examining the efficacy of a bimodal prehabilitation strategy that combines ß-lactoglobulin supplementation and resistance exercise training in modulating muscle protein metabolism following a short-term period of muscle disuse. As proof-of-concept, we hypothesise that this complex, short-term, non-pharmacological prehabilitation intervention will diminish the decline in MPS induced by 5 days of leg immobilisation in physically-active young males and females. To translate these data into clinical practice, follow up studies in more compromised populations are warranted, including patients due to undergo hip or knee replacement surgery [10, 11].

## Trial status

Protocol version 1 dated 15 December 2022. Recruitment start date: 1 March 2023. Recruitment end date: January 10, 2024.

## Supplementary Information

**Additional file 1.** SPIRIT checklist.

**Additional file 2.** Participant information sheet.

**Additional file 3.** Written informed consent.

## Data Availability

Yes

## Abbreviations

1-RM: One-repetition maximum
ANCOVA: Analysis of covariance
AUC: Area under the curve
CSA: Cross-sectional area
DXA: Dual-energy x-ray absorptiometry
ISAK: International Society of Anthropometry and Kinanthropometry
MPS: Muscle protein synthesis
