# Supplementary material for: The effect of combined β-lactoglobulin supplementation and resistance exercise training prior to limb immobilisation on muscle protein synthesis rates in healthy young adults: study protocol for a randomised controlled trial
Source: Trials. 2023 Jun 13;24:401. doi: 10.1186/s13063-023-07329-6 (PMC10265785; doi:10.1186/s13063-023-07329-6)
Supplement: Supplementary file 2 — Additional file 2. Participant information sheet. [file 13063_2023_7329_MOESM2_ESM.docx]

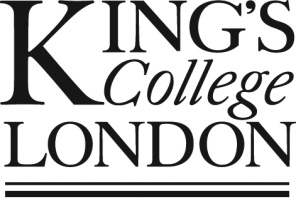
**INFORMATION SHEET FOR PARTICIPANTS**

*Ethical Clearance Reference Number: HR/DP-21/22-29290*

**YOU WILL BE GIVEN A COPY OF THIS INFORMATION SHEET**

**Title of project**

Impact of a milk protein supplement on muscle synthesis rates during resistance training and knee immobilisation in healthy young volunteers.

**Invitation Paragraph**

I would like to invite you to participate in this research on the impact of a milk protein supplement – β-lactoglobulin – on muscle synthesis rates during knee immobilisation. Muscle synthesis means the rate at which the muscle is able to make new muscle proteins. This study is a randomised controlled trial meaning that you will be assigned either the experimental drink (β-lactoglobulin) or the control (carbohydrate) drink. This study is being conducted as part of PhD degree at King’s College London. Before you decide whether you want to take part, it is important for you to understand why the research is being done and what your participation will involve. Please take time to read the following information carefully and discuss it with others if you wish. Ask me if there is anything that is not clear or if you would like more information.

**What is the purpose of the project?**

It is known that during periods of immobilisation such as injury, surgery, or bed rest, that there is a decrease in muscle synthesis rates that lead to reductions in muscle size and strength. These changes in muscle can have a detrimental impact on muscle function during recovery from injury. Some interventions such as protein supplements and exercise can prevent some of these associated changes. However, no research has investigated the impact of a combined exercise and nutrition regimen before a period of planned disuse and the impact this has on muscle synthesis. The purpose of this research is to see if a β-lactoglobulin supplement plus resistance exercise training for one week before a period of limb immobilisation will impact the decline in muscle synthesis that we see after disuse. This research will provide valuable information on interventions to prevent the decline in muscle synthesis that will help to establish strategies to promote recovery following injury and surgery.

**Why have I been invited to take part?**

You are being invited to participate in this project because you are an active individual between the age of 18-35 years. Participants should be active, completing at least 150–300 minutes of moderate-intensity aerobic physical activity per week OR 75–150 minutes of vigorous-intensity aerobic physical activity per week. Participants will not be asked to take part if they are sedentary (completing less than 150–300 minutes of moderate-intensity aerobic physical activity per week OR 75–150 minutes of vigorous-intensity aerobic physical activity per week), take part in structured resistance exercise training, have had a lower limb injury or surgery in the past 6 months, have lower limb osteoarthritis or other musculoskeletal disorder, are currently pregnant, using blood thinning medications, have a blood clotting disorder, have an allergy to dairy or local anaesthetic, or are currently taking supplements known to influence muscle such as protein, creatine, or omega-3s/fish oils. You will also be asked to not take part if you have taken part in another study using a stable isotope tracer in the past 18 months. For female participants, you should have a regular menstrual cycle, not be taking a hormonal contraceptive, and not have taken a form of hormonal contraception in at least a year. A regular menstrual cycle includes a cycle lasting between 21-40 days, with no missed periods in the past year. Females who do not menstruate for any reason will not be included as it will be difficult to determine which phase of your cycle you are in throughout the study.

**What will happen if I take part?**

If you choose to take part in the project you will be asked to attend our laboratories on 8 separate occasions. Five of these sessions will involve testing, and four will involve monitored resistance exercise training, with some overlap between the two.

Prior to attending the first laboratory session, you will be provided with a three-day food diary and a pair of food scales to take home with you. These will be used to assess your baseline dietary intake before you embark on the study. This will involve weighing and writing down your food intake for three days, including at least one weekend day. This baseline dietary data will be used to determine if you are eligible to take part in the study. It may be the case that after completing this you are not deemed eligible to complete the study and will not be invited to take part. It is worth noting that individuals with a history of eating disorders may find tracking food intake and weighing food distressing. If you believe this may be the case you may wish to not take part in this study, although this is entirely your decision. You will then be randomised to either the milk protein supplement group or the carbohydrate placebo. The lead investigator will then randomise choice of leg to be immobilised using the same randomisation spreadsheet, such that 50% of participants have their dominant leg immobilised and 50% of participants their non-dominant leg immobilised.

On the first session, you will be provided with information about the trial and will be asked to sign a consent form if you agree to take part. We intend on taking your contact details, and details of your doctor’s surgery in the unlikely event that any issues arise from the period of leg immobilisation, i.e., deep vein thrombosis. During the first session we will measure your height and weight. Height and weight will also be measured during the follow up sessions that you have after the resistance exercise training period, as well as after the immobilisation period. A baseline blood sample will be taken (approximately 14ml of blood), as well as a saliva sample. You will also be given a labelled water beverage (heavy water). You will be given 3ml/kg of body mass to drink in 50ml doses separated by 30 minutes. This water will be consumed in the lab. We will also assess your body composition using a technique called skinfolds. The researcher will pinch sections of your skin and fat and measure the thickness using a special calliper. The first testing session will involve an assessment of your one repetition maximum (1RM) for single leg press and leg extension. You will be asked to lift as much weight as possible in one maximal effort attempt for each exercise. You will also be familiarised with the strength assessment. For this you will be seated on a purpose-built chair with a brace above your ankle. You will be asked to contract your thigh muscles as hard as possible under dynamic and static conditions. For the dynamic condition you will be asked to contract your muscle to bring your leg from a 90-degree angle to straight, and back again. For the static condition, you will be asked to contract your muscles maximally while your leg is kept at 90 degrees. You will be asked to perform three maximal effort attempts and be asked to sustain these for 3 seconds. During this test, we will also measure voluntary activation of your muscle using nerve stimulation (electrical stimulation of leg muscles during contraction for measurement of maximal force). This will involve placing two electrodes over the belly of your thigh muscle. Single electrical impulses will be delivered whilst you contract your muscle. These procedures may feel strange or uncomfortable as the stimulation will make your muscles contract and leg move without you doing anything. Finally, you will be asked to provide another blood and saliva sample, and you will be provided with top up doses of the heavy water drink to take home with you and consume every day for the remainder of the study period. You will be asked to take daily saliva swabs prior to taking this drink each day, for which you will be provided saliva sampling kits. You will also be given a device and a questionnaire that will monitor your physical activity of the next three days. You will be asked to wear this device each day, and take it off at night for sleep, for three days, up until your next visit to the lab. You will be asked to fill out the questionnaire for these three days as well. This questionnaire will assess your physical activity levels every 15 minutes, and you will be asked to fill this out throughout the day as frequently as possible. This may involve keeping the questionnaire with you and being aware of the types of activity you are performing on a day-to-day basis. The device will be worn, and the questionnaire completed, two other times throughout the study period, for three days each time.

Your next visit will take place three days later. During this session an ultrasound of your thighs will be taken. This will involve an imaging probe being placed on your skin which will be coated in a water-based gel. At this time we will also measure the circumference of your thigh and calf muscle on each leg. We will then assess the strength of your quadriceps and hamstrings using the same purpose-built chair used in your familiarisation session. Finally, a laboratory testing session will take place where you will have a sample of muscle taken from the outside of your thigh. This procedure involves cleaning the skin and numbing the area with a local anaesthetic via injection. Once the area is numb, a small incision (approximately 0.5cm) will be made through which a needle will be inserted. The biopsy needle will be put into the muscle and a few pieces collected (about the size of a pea). The needle is removed and the sample frozen. While the sample is being taken the main feeling people experience is a small amount of pressure. After the sample has been taken pressure will be applied to the area to stop any bleeding. Steri-strips (i.e. thin adhesive strips) will be applied to close the site. A large plaster and waterproof dressing will be put over the site and you will need to keep this dry for 48 hours.

Muscle tissue from biopsy samples will be used to assess the rate at which your muscle proteins are being made, known as muscle protein synthesis. They will also be used to look at markers of muscle protein breakdown, as well as the size of your muscle fibres and your muscle fibre type.

After this initial assessment, you will perform four exercise training sessions over the course of the next week. These sessions will take place at the Centre for Human and Applied Physiological Sciences and will be monitored by a researcher. Each session will consist of two warm-up sets at 50% of 1RM, followed by six sets at 75% of 1RM for both the single leg press and single leg extension. Sets will consist of a target 12 repetitions and will be separated by 2 minutes of rest. During the middle one of these sessions, another blood sample will be taken.

After this training period, the same testing session described above will be repeated. You will then have one leg (randomly decided) immobilised with a leg brace and boot. The brace is designed to maintain the knee joint at a 90֯ angle for 5 days. You will be instructed on the use of crutches for ambulation during this period, and also provided with a waterproof cover to put over the boot and brace when showering. After this 5-day period, the brace will be removed, and the above measurements repeated for a third time. After this you are advised to complete the training protocol provided for you in the participant advice sheet, although this will not be monitored.

Throughout the trial, following the first full testing day, you will be randomised to drink either a milk protein supplement (β-lactoglobulin) or a placebo (dextrose monohydrate) for the entire trial period. You will not be aware of which drink you are taking throughout the study period. You will be asked to drink this every day, three times per day, with each meal. You will be provided with pre-packaged amounts of the protein or placebo to mix with water at each meal. For the β-lactoglobulin each dose will be 23grams. For the placebo, an equivalent, calorie matched amount will be provided.

Participation will take place on Guy’s Campus at the Centre for Human and Applied Physiological Sciences.

A detailed schematic of the protocol can be seen below:


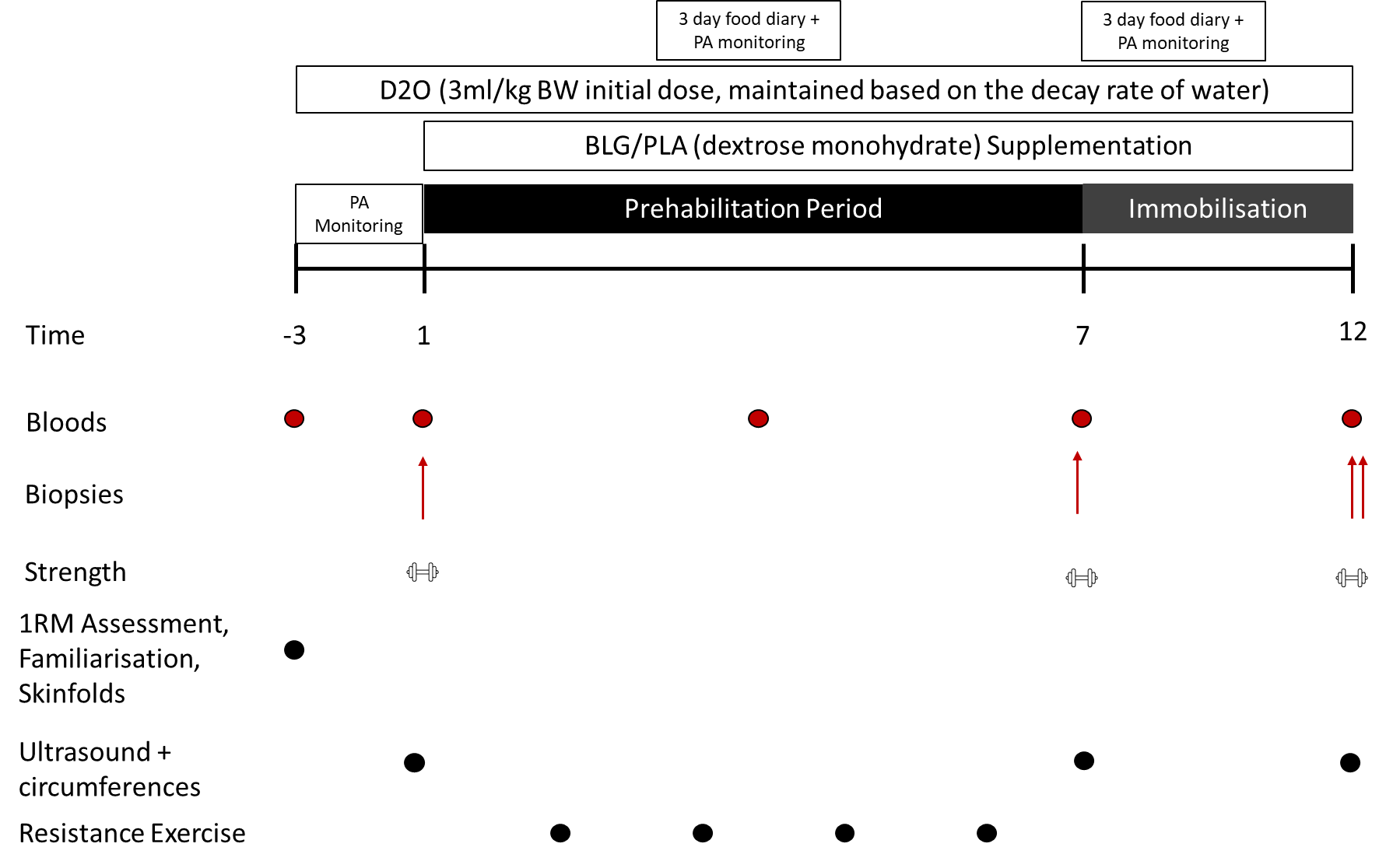


**Female Participants:**

Female participants will be asked to track the start and end of their menstrual cycles for a total of three months – one cycle prior to taking part in the study, one cycle throughout the study period, and one cycle after the study has ended. This tracking will be done by simply reporting to the researchers the date that your menstrual cycle starts (i.e., the first day of your period) at each of these time points. You will be asked to begin the first day of the study (the first lab day after familiarisation) as close to the start of your menstrual cycle as possible. This will be discussed with you when scheduling in your testing dates. These data will be collected by Miss A Hughes.

**Do I have to take part?**

Participation is completely voluntary. You should only take part if you want to, and choosing not to take part will not disadvantage you in any way. Once you have read the information sheet, please contact us if you have any questions that will help you make a decision about taking part. If you decide to take part, we will ask you to sign a consent form and you will be given a copy of this consent form to keep.

**Incentives**

You will be reimbursed for any travel costs you incur while taking part in the study up to a £75 maximum. You will also receive £500 for full participation in the study. This amount will be paid at the end up of the study. If you decide to drop out of the study after the first lab testing day (day 1 on the flowchart), you will receive £166. If you decide to drop out after the second lab testing day (day 7 on the flowchart), you will receive £332.

**What are the possible risks of taking part?**

For the muscle biopsies, a sterile non-disposable Bergstrom needle will be used. This is a standard piece of equipment for taking muscle samples for research and clinical practice. A very stringent sterilization procedure is in place which involves multiple rounds of cleaning, disinfection, and sterilization. You may feel a string when the anaesthetic is first put in, but you will not feel the incision. You will feel a sensation of pressure when the needle is inserted. When the anaesthetic has worn off you may see some bruising and you may feel as though you have knocked your leg on the corner of a table. To avoid any discomfort and to make sure the area heals properly, you are advised to avoid any vigorous activity until the soreness subsides – usually within a few days. Risk of infection is minimized by keeping the wound dry for at least 48 hours. Swelling can be reduced by elevating the limb and applying an ice pack. You will be given a phone number so that you can contact a member of the research team if you have any concerns. If required, you can come back to King’s and a member of the research team can check the steri-strips. In the unlikely event that one of the team is not available and there is bleeding or serious discomfort you should contact your GP or go to the local hospital.

Blood samples will be taken during visits to the lab, which carries the risk of discomfort and potential bruising around the site of sampling. Blood samples will be taken by experienced and trained phlebotomists who will take appropriate precautions to minimize this risk. There is also a slight risk of fainting during or following blood sampling. To reduce this risk, participants will be on a semi-reclined physiotherapist bed throughout the duration of the sampling and for five minutes following sampling. A researcher will be always present with the participant to ensure no falls or injuries are sustained in the event of fainting.

If you take part in this study, you will have an ultrasound scan taken of your thighs. These scans have the potential to detect abnormal physiology, such as masses or musculoskeletal issues if they are present. In the highly unlikely event that any adverse or incidental findings are made from the data we collect from you, the principal investigator will discuss this with you. If necessary, we will contact your GP with a report detailing the findings.

This research involves taking a labelled water drink in order to assess muscle synthesis rates. Very low doses of this drink will be given to you, and it has been used extensively in other research safely. However, there is a small possibility that you may develop some side effects of taking this drink. These include nausea, dizziness, and light-headedness. These side effects usually only occur with high doses (>300ml) of the drink, and the dosing strategy has been selected in order to reduce this risk.

This research involves a five-day period of limb immobilisation, which is associated with a loss of muscle mass. It is expected that the individuals taking the protein supplement will have less muscle loss than those taking the placebo, although this is not guaranteed. Periods of immobilisation are also a risk factor for deep vein thrombosis. While this is not expected in young, healthy individuals, it is a possibility as part of taking part in this research. You will be provided with an advice sheet to inform you of signs and symptoms to look out for during the study, as well as advice on the other possible adverse events mentioned in this information sheet.

In light of the current Covid-19 pandemic, all researchers will be wearing face masks during testing and the building in which testing takes place in has a one-way system to reduce contact between individuals.

**What are the possible benefits of taking part?**

Participants will have the opportunity to have their muscle volume and muscle strength tested under laboratory conditions, providing them a greater understanding of their overall physical health. We also believe that taking β-lactoglobulin supplementation will have benefits to muscle mass, strength, and function. Therefore, the participants in the β-lactoglobulin group may experience these health benefits, although this is not guaranteed.

**Data handling and confidentiality**

Your data will be processed under the terms of UK data protection law (including the UK General Data Protection Regulation (UK GDPR) and the Data Protection Act 2018). Each participant will be allocated a specific identification number which will be used to refer to the participant for all of their data. This participant identification number will keep the data pseudonymous and maintain participant confidentiality. Anonymity will be maintained in any published report, which will not include any identifiable information of any participants. Data will be stored securely (on King’s College London OneDrive servers on password protected laptops in the PhD student’s office and the principal investigator’s office) and will only be accessible to the researchers on this project. The researchers on this project only include individuals from the research team in King’s College London, researchers at the University of Nottingham, and researchers at the University of Birmingham. Data generated from your tissue (saliva, blood and muscle) samples will be shared between KCL and Nottingham and Birmingham. No personal data (i.e., age, sex, body mass, etc) will be shared with Nottingham or Birmingham. After the study has been completed on the 1st of January 2025 all data, aside from participant contact details, will be fully anonymized and stored for up to 5 years, after which time all data will be destroyed. Participant contact details will be kept so that you may be contacted about the outcome of the study and any publications, as well as being contacted about other studies that you may be interested in taking part in. Any physical forms, such as the consent forms, will be held in a locked filing cabinet in a locked room at the Centre for Human and Applied Physiological Sciences at King’s College London.

**Data Protection Statement**

If you would like more information about how your data will be processed under the terms of UK data protection laws please visit the link below:

<https://www.kcl.ac.uk/research/support/research-ethics/kings-college-london-statement-on-use-of-personal-data-in-research>

**What if I change my mind about taking part?**

You are free withdraw at any point of the project, without having to give a reason. Withdrawing from the project will not affect you in any way. You are able to withdraw your data from the project up until 1^st^ of January 2025 after which withdrawal of your data will no longer be possible as the data will have been anonymised and committed to the final report. If you choose to withdraw from the project, we will not retain the information you have given thus far.

**How is the project being funded?**

This project is being funded by the Medical Research Council Doctoral Training Partnership and a King’s Health Partners Challenge Fund. Supplements will be provided in kind by Arla Food Ingredients. Arla Food Ingredients will not have any access to the data collected as part of this study.

**What will happen to the results of the project?**

The results of the project will be summarized in a published article and presented at national and international conferences. Please request if you wish to obtain a copy of the published research.

**Who should I contact for further information?**

If you have any questions or require more information about this project, please contact me using the following contact details:

Miss Alix Hughes: [alix.hughes@kcl.ac.uk](mailto:alix.hughes@kcl.ac.uk)

Dr Oliver Witard (PI): [oliver.witard@kcl.ac.uk](mailto:oliver.witard@kcl.ac.uk)

Department Postal Address (address to either of the above names):

Centre for Human and Applied Physiological Sciences

Shepherd’s House

SE1 1UL

London

**What if I have further questions, or if something goes wrong?**

If this project has harmed you in any way or if you wish to make a complaint about the conduct of the project you can contact King's College London using the details below for further advice and information:

Dr Oliver Witard: [oliver.witard@kcl.ac.uk](mailto:oliver.witard@kcl.ac.uk)

Department Postal Address:

Centre for Human and Applied Physiological Sciences

Shepherd’s House

SE1 1UL

London
