# Supplementary material for: The effect of combined β-lactoglobulin supplementation and resistance exercise training prior to limb immobilisation on muscle protein synthesis rates in healthy young adults: study protocol for a randomised controlled trial
Source: Trials. 2023 Jun 13;24:401. doi: 10.1186/s13063-023-07329-6 (PMC10265785; doi:10.1186/s13063-023-07329-6)
Supplement: Supplementary file 3 — Additional file 3. Written informed consent. [file 13063_2023_7329_MOESM3_ESM.docx]

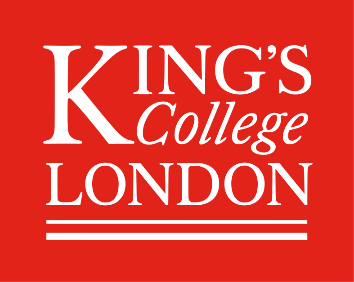
**CONSENT FORM FOR PARTICIPANTS IN RESEARCH PROJECTS**

Please complete this form after you have read the Information Sheet and/or listened to an explanation about the research

| **Title of project:** Impact of a milk protein supplement on muscle protein metabolism during resistance training and knee immobilisation in healthy young volunteers | | |
| --- | --- | --- |
| **Ethical review reference number: HR/DP-21/22-29290** | **Version number: 30/01/2023 version 7** | |
|  | | Tick or initial |
| 1. I confirm that I have read and understood the information sheet dated 30/01/23, version 9 for the above project. I have had the opportunity to consider the information and asked questions which have been answered to my satisfaction. | |  |
| 1. I consent voluntarily to be a participant in this project and understand that I can refuse to take part and can withdraw from the project at any time, without having to give a reason, up until 1^st^ of January 2025. | |  |
| 1. I consent to the processing of my personal information for the purposes explained to me in the Information Sheet. I understand that such information will be handled under the terms of UK data protection law, including the UK General Data Protection Regulation (UK GDPR) and the Data Protection Act 2018. | |  |
| 1. I understand that my information may be subject to review by responsible individuals from the College for monitoring and audit purposes. | |  |
| 1. I understand that confidentiality and anonymity will be maintained, and it will not be possible to identify me in any research outputs. | |  |
| 1. I agree that the research team may use my data for future research and understand that any such use of identifiable data would be reviewed and approved by a research ethics committee. (In such cases, as with this project, data would not be identifiable in any report). | |  |
| 1. I acknowledge and agree to allow the transfer of my muscle, blood and saliva samples to the University of Nottingham, for analysis of muscle and metabolic responses. | |  |
| 1. I acknowledge and agree to allow the transfer of my muscle to the University of Birmingham for analysis of muscle and metabolic responses. | |  |
| 1. I consent for The Universities of Nottingham and Birmingham to have access to my anonymised personal data. | |  |
| 1. I understand that I must not take part if I fall under the exclusion criteria as   detailed in the information sheet and explained to me by the researcher. | |  |
| 1. I understand that the information I have submitted will be published as a report. | |  |
| 1. I wish to receive a copy of the final report. | |  |
| 1. I agree to be re-contacted in the future by King’s College London researchers regarding this project. | |  |
| 1. I agree that the researcher may retain my contact details so that I may be contacted in the future by King’s College London researchers who would like to invite me to participate in future studies of a similar nature. | |  |
| 1. I consent to the collection of (84ml) of my blood over the course of the study. | |  |
| 1. I consent to the collection of approximately 1500mg of my quadriceps muscle over the course of the study. | |  |
| 1. I consent to participate in the use of an ultrasound scan. | |  |
| 1. I consent to the collection of daily saliva samples throughout the course of the study. | |  |
| 1. I consent to the use of a Lidocaine injection into the skin and muscle of my quadriceps to numb the area prior to the collection of the muscle sample. | |  |
| 1. I consent to the completion of a food frequency and physical activity questionnaire. | |  |
| 1. I consent to participation in the assessments of muscle strength. | |  |
| 1. I consent to peripheral nerve stimulation of my leg muscles. | |  |
| 1. I consent to my body composition being assessed via skinfold measurements | |  |
| 1. I agree that my GP may be contacted if any unexpected results are found in relation to my health (please provide GP contact details in the space below). | |  |
| 1. I consent to my data from this study being used towards an NHS REC approved study (IRAS: 317347) | |  |

**GP contact details**

| GP Practice Name: | GP Name: |
| --- | --- |
| Practice Phone Contact: | GP Email Contact: |
| Practice Postal Address: | |

**__________________ __________________ _________________**

**Name of Participant Date Signature**

**__________________ __________________ _________________**

**Name of Researcher Date Signature**
